# ESTRO-EPTN radiation dosimetry guidelines for the acquisition of proton pencil beam modelling data

**DOI:** 10.1016/j.phro.2024.100621

**Published:** 2024-08-05

**Authors:** Carles Gomà, Katrin Henkner, Oliver Jäkel, Stefano Lorentini, Giuseppe Magro, Alfredo Mirandola, Lorenzo Placidi, Michele Togno, Marie Vidal, Gloria Vilches-Freixas, Jörg Wulff, Sairos Safai

**Affiliations:** a Institute of Cancer and Blood Diseases, Hospital Clínic Barcelona, Barcelona, Spain; b Translational Genomics and Targeted Therapies in Solid Tumors, August Pi i Sunyer Biomedical Research Institute (IDIBAPS), Barcelona, Spain; c Catalan Health Service, Barcelona, Spain; d Heidelberg Ion Beam Therapy Center at the Heidelberg University Hospital, Heidelberg, Germany; e German Cancer Research Center (DKFZ), Heidelberg, Germany; f Medical Physics Department, Azienda Provinciale per i Servizi Sanitari (APSS), Trento, Italy; g Medical Physics Unit, National Center for Oncological Hadrontherapy (CNAO), Pavia, Italy; h Fondazione Policlinico Universitario Agostino Gemelli, IRCCS, Department of Diagnostic Imaging, Oncological Radiotherapy and Hematology, Rome, Italy; i Center for Proton Therapy, Paul Scherrer Institute, Villigen, Switzerland; j Institut Méditerranéen de Protonthérapie - Centre Antoine Lacassagne, Nice, France; k Department of Radiation Oncology (Maastro), GROW School for Oncology and Reproduction, Maastricht University Medical Centre, Maastricht, The Netherlands; l West German Proton Therapy Centre Essen (WPE), Essen, Germany; m University Hospital Essen, Essen, Germany

**Keywords:** Proton therapy, Radiation dosimetry, Pencil beam scanning, Beam modelling, Guidelines

## Abstract

Proton therapy (PT) is an advancing radiotherapy modality increasingly integrated into clinical settings, transitioning from research facilities to hospital environments. A critical aspect of the commissioning of a proton pencil beam scanning delivery system is the acquisition of experimental beam data for accurate beam modelling within the treatment planning system (TPS). These guidelines describe in detail the acquisition of proton pencil beam modelling data. First, it outlines the intrinsic characteristics of a proton pencil beam—energy distribution, angular-spatial distribution and particle number. Then, it lists the input data typically requested by TPSs. Finally, it describes in detail the set of experimental measurements recommended for the acquisition of proton pencil beam modelling data—integrated depth-dose curves, spot maps in air, and reference dosimetry. The rigorous characterization of these beam parameters is essential for ensuring the safe and precise delivery of proton therapy treatments.

## 1 Introduction

Proton therapy (PT) is a radiotherapy technique that is becoming increasingly accessible, with new facilities being built in hospitals rather than research facilities. As such, newcomers to PT are typically well-trained healthcare professionals with an advanced knowledge of conventional radiotherapy and a limited knowledge gap specific to PT. One of the missions of the European Particle Therapy Network (EPTN) is to contribute to narrow this gap with expertise and consensus from established European PT centres [1], [2], [3], [4].

From a medical physics point of view, the commissioning of a proton pencil beam scanning (PBS) delivery system is critical. Thus far, several publications have reported different user experiences [5], [6], [7], [8], [9], [10], [11], but it was not until 2021 that the first generic document, the AAPM TG-185 report [12], was published. This report is a valuable overview document that covers all the relevant aspects of proton PBS machine commissioning. However, many of these aspects—such as IGRT, SGRT or motion management—are not unique to PT. On the other hand, it lacks detail in points that are specific to PT, such as the acquisition of proton pencil beam modelling data.

The aim of these guidelines is therefore to describe in detail the acquisition of proton pencil beam modelling data and to provide a radiation dosimetry handbook for experienced radiotherapy medical physicists who are newcomers to the field of PT.

## 2 Scope and terminology

These guidelines cover proton PBS delivery systems only. It focuses on the beam data input typically requested by treatment planning systems (TPSs) for the modelling of proton PBS machines. As a general rule, these guidelines do not cover beam data validation. However, some validation tests will be mentioned when the experimental measurements needed are the same as (or very similar to) those needed for beam data acquisition. Quality assurance [13] falls beyond the scope of these guidelines.

Regarding treatment delivery systems (TDSs), these guidelines intend to be generic (i.e. not vendor-specific) and cover all TDSs currently installed in Europe. As for treatment planning systems, it covers the requirements of the most widespread systems in Europe.^12^

The proton-specific terminology used in these guidelines is described in Table 1. The guidelines initially describe the intrinsic characteristics of a proton pencil beam. Second, it explains how to determine these characteristics experimentally. Third, it lists the input data typically requested by TPSs, as this data does not always match the experimental measurements described previously. Finally, based on the input data requested by TPSs, it describes in detail the set of experimental measurements recommended for the acquisition of proton pencil beam modelling data.

**Table 1:** Proton-specific terminology.

**Term (recommended)**	**Abbreviation/acronym**	**Example** **units**	**Definition**	**Alternative terms**
Angular-spatial distribution	ASD	rad^−1^ cm^−1^	The angular and spatial distribution of a proton pencil beam may be described—independently for the two axes (*x* and *y*) perpendicular to the beam propagation axis (*z*)—using a bivariate Gaussian distribution of the position (*x*) and the angle (θ): $ASD\left(x,\theta ,z\right)=\frac{e^{-\left(\frac{\sigma_{\theta }^{2}x^{2}-2\sigma_{x\theta }x\theta +\sigma_{x}^{2}\theta^{2}}{\sigma_{\theta }^{2}\sigma_{x}^{2}-\sigma_{x\theta }^{2}}\right)}}{2\pi \sqrt{\sigma_{\theta }^{2}\sigma_{x}^{2}-\sigma_{x\theta }^{2}}}$ where $\sigma_{x}^{2}$,$\sigma_{x\theta }$ and $\sigma_{\theta }^{2}$ are the moments of the distribution in the (*x*,θ) phase space plane. In vacuum, these may be expressed as a function of *z* as: $$\sigma_{\theta }^{2}\left(z\right)=\sigma_{\theta }^{2}\left(0\right)\;\sigma_{x\theta }\left(z\right)=\sigma_{\theta }^{2}\left(0\right)z+\sigma_{x\theta }\left(0\right)\;\sigma_{x}^{2}\left(z\right)=\sigma_{\theta }^{2}\left(0\right)z^{2}+2\sigma_{x\theta }\left(0\right)z+\sigma_{x}^{2}\left(0\right)$$ In matter, a scattering term must be added to each of these equations [17], resulting in the generalised Fermi-Eyges theory.	Beam optics
Bragg peak			Distal part of the integrated depth-dose curve where the integrated absorbed dose reaches its maximum value.	
Dose-area product	DAP	Gy cm^2^	Integral of the absorbed dose over the plane perpendicular to the beam propagation axis.	Integrated absorbed dose
Energy spread	σ_E_	MeV	Standard deviation of the energy distribution of the beam.	
Entrance region			Proximal part of the integrated depth-dose curve.	Plateau region [18]
Integrated depth-dose curve	IDD		Integral of the absorbed dose over the plane perpendicular to the beam propagation axis as a function of the depth in the medium. That is, DAP as a function of depth.	Integral depth-dose (curve)
Large-area ionization chamber	LAIC		Plane-parallel ionization chamber with a sensitive volume diameter equal to or larger than 8 cm.	Bragg peak chamber, large-diameter ionization chamber
Nozzle			Last element of the beamline, which houses the beam monitors and houses/holds the beam accessories (e.g. range-shifters and apertures).	
Mean energy	E	MeV	Mean value of the energy distribution of the beam.	
Particle number	N		Number of particles that are emitted, transferred or received [19].	Number of protons
Peak-to-entrance ratio			Ratio between the maximum of the IDD curve and the value at the entrance region.	Peak-to-plateau ratio
Pencil beam			A radiation beam with a finite (but small) initial ASD and a finite (but small) initial energy spread [20].	Finite pencil beam, beamlet
Pencil beam scanning	PBS		Beam delivery technique based on the scan of proton pencil beams using magnetic fields.	Spot scanning, discrete scanning
Range	*R_80_*	cm	Depth in the medium at which the integrated absorbed dose beyond the Bragg peak falls to 80 % of its maximum value.	
Range-shifter			Uniform slab of material inserted in the beam to reduce the mean energy (and range) of the beam. It is typically used to treat shallow-seated targets.	Energy absorber, pre-absorber
Source-axis distance	SAD	cm	Distance between the isocentre and the effective point of deflection of the beam in the scanning magnets.	
Spot			A proton pencil beam with a specific energy and lateral position.	
Spot profile			One-dimensional dose distribution of a proton pencil beam along one axis orthogonal to the beam propagation axis.	
Spot shape			Two-dimensional dose distribution of a proton pencil beam in the plane orthogonal to the beam propagation axis.	
Spot size	(σ_x_, σ_y_)	cm	Width of the spot profile/shape, typically characterized by the standard deviation (σ) of the spatial distribution along the two axis orthogonal to the beam propagation axis (σ_x_, σ_y_).	
Spot map			The simultaneous experimental measurement of the spot shape at different lateral positions, typically following a regular pattern.	
Spot spacing	Δ	cm	Distance between the centre of adjacent spots.	

## 3 Intrinsic characteristics of a proton pencil beam

A clinical proton pencil beam^13^ may be described by three intrinsic characteristics, its energy distribution, angular-spatial distribution (ASD) and the particle number, as well as a geometrical characteristic of the TDS, the source-axis distance (SAD). We typically refer to the *initial* energy distribution, *initial* ASD and *initial* particle number to specify these characteristics in air and at isocentre.

**Fig. 1:**
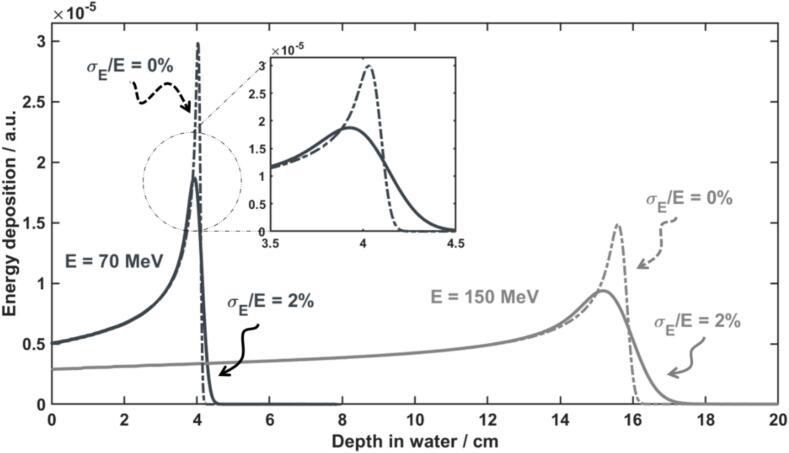
Integrated depth-dose curves of proton pencil beams of different initial mean energy (70 and 150 MeV) and relative energy spread of 0 % (dashed lines) and 2 % (solid lines).

The energy distribution of a proton pencil beam is for most TDSs well represented by a Gaussian distribution. Thus, it can be described by its mean value (mean energy, *E*) and the standard deviation (energy spread, *σ_E_*). The initial mean energy of the beam is very well correlated with the range of the beam in the medium (*R_80_*) [21], [22]. The initial energy spread of the beam determines the sharpness of the Bragg peak in depth, as well as the peak-to-entrance ratio—see Fig. 1.

**Fig. 2:**
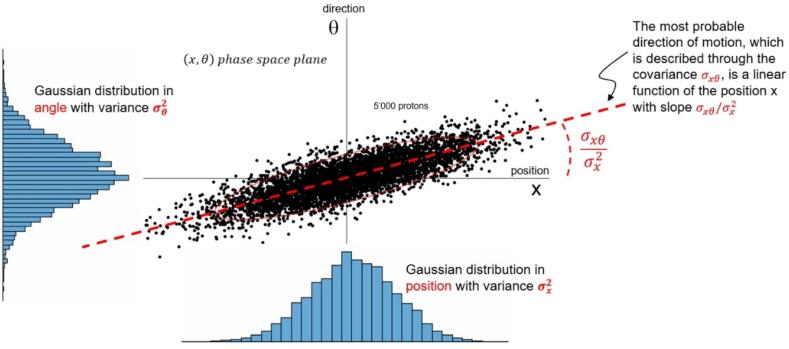
Phase space for 5000 protons randomly distributed following a bivariate Gaussian distribution in *x* and θ, with the moments of the angular-spatial distribution ($\sigma_{x}^{2}$, $\sigma_{x\theta }$ and $\sigma_{\theta }^{2}$) explained. The isolines of the distribution in the (x,θ) plane are ellipses. As the beam propagates in vacuum, the ellipses change their shape, but the number of particles inside the ellipses remains constant.

The angular-spatial distribution (ASD) refers to the *shape* (spatial distribution) and *intrinsic divergence* (angular distribution) of a proton pencil beam. Mathematically, it can be described, independently for the two axes (*x* and *y*) perpendicular to the beam propagation axis (*z*), using a bivariate Gaussian distribution of the position (*x*) and the angle (θ)—see Table 1. The parameters of the distribution—spatial variance ($\sigma_{x}^{2}$), angular variance ($\sigma_{\theta }^{2}$) and covariance ($\sigma_{x\theta }$)—are referred to as the *moments* of the ASD in the (*x*,θ) phase space plane and are depicted in Fig. 2. Of particular interest for the determination of these moments is the description of the spatial variance along the beam propagation axis, which may be expressed as [23]:

$$\sigma_{x}^{2}\left(z\right)=\sigma_{x}^{2}\left(0\right)+2\sigma_{x\theta }\left(0\right)z+\sigma_{\theta }^{2}\left(0\right)z^{2}$$

where $\sigma_{x}^{2}\left(0\right)$, $\sigma_{\theta }^{2}\left(0\right)$ and $\sigma_{x\theta }\left(0\right)$ are the initial moments of the ASD (i.e. at z=0). An equivalent equation holds true for the (*y*,θ) phase space plane. The additive scattering term [17]—which leads to the generalized Fermi-Eyges theory—is typically ignored for the beam propagation in air, but it should be considered in the patient.

Furthermore, in a clinical treatment delivery system, the particle number is controlled by the beam monitor chambers. The particle number per monitor unit (MU)—defined here as the *output* of the TDS—is a function of (i) the mean energy of the beam, and (ii) the beam divergence—which increases the track length in the monitor chamber. This latter beam divergence effect, however, is directly correlated with the lateral position of the beam and it is typically corrected for internally in the TDS. Thus, in practical terms, the user only needs to determine the functional dependency of the output with respect to the mean energy of the beam in the nozzle axis.

Finally, although it is not strictly an intrinsic characteristic, magnetically-scanned proton pencil beams are also described by an additional geometric characteristic of the TDS: the source-axis distance (SAD)—which is the distance between the isocentre and the effective point of deflection of the beam in the scanning magnets. Note that this distance is typically different for *x* and *y*.

## 4 Experimental determination of proton pencil beam intrinsic characteristics

### 4.1 Integrated depth-dose curves in water

The integrated depth-dose (IDD) curve in water of a single proton pencil beam is used to determine its initial energy distribution, in particular the initial mean energy (*E*) and the initial energy spread (*σ_E_*). The initial mean energy can be derived directly from the range (*R_80_*) in water [21]. The initial energy spread of the beam is typically determined via an iterative process that minimizes the differences between the experimental and computed IDD. This is typically done internally in the TPS.

### 4.2 Spot shape and spot maps in air

The spot shape is the two-dimensional dose distribution of a proton pencil beam in the plane orthogonal to the beam propagation axis (*xy*-plane). This full 2D information is needed as spots are not always symmetrical.

The initial moments of the ASD may be determined by the experimental measurement of the spot shape in air at different distances along the beam propagation axis (*z*-axis). Since the spatial variance ($\sigma_{x}^{2}$) may be described as a 2nd degree polynomial of *z* (see Eq. (1)), at least three data points at different *z*-positions are needed to extract the initial moments of the ASD by means of a least-squares fit.

**Fig. 3:**
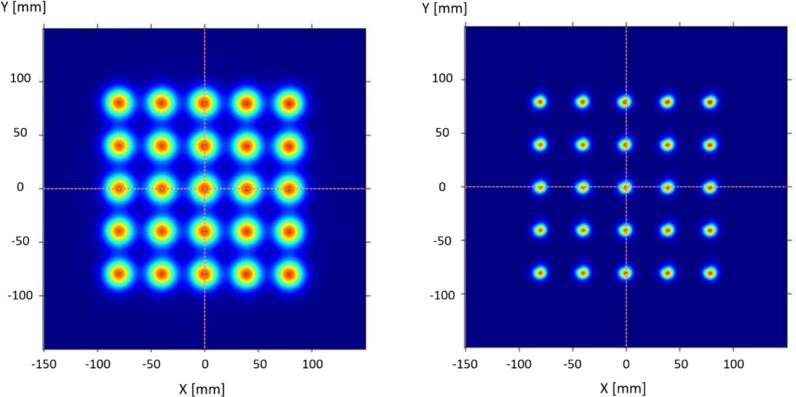
Spot maps in air at isocentre for two different energies (154 and 227 MeV) in a Mevion 250i HYPERSCAN delivery system.

A spot map (see Fig. 3) is the experimental measurement of the spot shape at different lateral positions of the beam. Although they are not strictly necessary for the determination of the initial moments of the ASD, spot maps are typically preferred to single spot shape measurements, since the spot shape may depend on the lateral position of the beam (and the gantry angle, as discussed below). Furthermore, spot maps at different *z*-positions also allow to determine the source-axis distance (SAD)—using the distance between spots together with simple triangle similarity—as well as to verify the lateral position of the beam.

### 4.3 Output measurement

An absolute determination of the particle number (per MU) could in principle be obtained by means of a Faraday cup [24], [25], [26], [27], [28], [29]. This detector, however, is not standard and the traceability to other external radiotherapy beams is difficult.

A much more widespread approach to obtain the particle number (per MU) is to determine the absorbed dose to water (*D_w_*) in the middle of a broad radiation field using a reference air-filled ionization chamber and following a radiation dosimetry protocol/code of practice [18]. This approach, called reference dosimetry, is the one recommended in these guidelines. If needed in the TPS, the absorbed dose to water at a point can be converted to particle number by means of a theoretical model [30]. This last step is typically needed for Monte Carlo (MC) dose calculation engines and it is performed internally in the TPS.

## 5 Treatment planning system input data requirements

Treatment planning systems typically ask for the following set of data. For a comprehensive set of proton beam energies—which includes the minimum and maximum beam energy deliverable by the TDS—they request:

• The integrated depth-dose curve—to determine internally the initial energy distribution of the beam.
• Lateral spot profiles in air at minimum three different *z*-positions—to determine the initial moments of the ASD.^14^
• The absorbed dose to water (per MU) in the middle of a broad field or the integrated absorbed dose to water (per MU) of a single pencil beam.

Regarding the beamline,^15^ they request:

• The source-axis distance (SAD).^16^
• The range-shifter(s) water-equivalent thickness (WET).^17^

## 6 Radiation dosimetry guidelines for TPS input data acquisition

Typically, TDSs are capable of delivering proton pencil beams ranging from 60-70 MeV to slightly above 230 MeV. As such, TPSs require dosimetric data for a comprehensive set of proton energies, typically in steps of 10 MeV, including the lowest and highest deliverable energies.

These guidelines advise adhering to these minimal TPS requirements. Specifically, this means acquiring data across the entire range from the lowest to the highest deliverable energies, in steps of 10 MeV. This recommendation assumes that beam optics vary smoothly with energy. If this assumption does not hold true, a finer energy resolution may be advisable.

One of the treatment delivery systems installed in Europe^18^ is notably different from the rest, in the sense that it generates a single high-energy beam (configurable up to 250 MeV) and the range is adjusted in the nozzle by means of a set of range-shifter plates. As such, only one single beam needs to be modelled. However, for validation purposes, beam data acquisition is recommended for at least 15 representative energies (i.e. combination of range-shifter plates), covering the interval of deliverable energies and chosen so that each plate is included at least once in combination with as few other plates as possible.

### 6.1 Integrated depth-dose curves in water

IDDs should be measured in water. The use of water-equivalent plastics is not recommended [18]. IDDs should be measured with a large-area ionization chamber [18]. The entrance window should be thick enough (WET ≥ 4 mm) to avoid bending due to water pressure. Ref. [31] reported a large lateral heterogeneity for 2 mm-wall large-area ionization chambers. The WET of the entrance window must be accounted for. Thus, manufacturer technical specifications should be accurate and precise, since any deviation from these technical specifications would introduce a systematic error in the determination of the range in water. If possible, the WET of the entrance window should be verified experimentally. If this is not possible (without damaging the detector), the user may verify it by comparative measurements with other radiation detectors.

These guidelines recommend the use of a reference signal to normalize the reading of the field chamber and correct for possible beam intensity fluctuations during the scan. When possible, it is advised to use the signal of the primary/secondary beam monitor chamber in the nozzle. If not possible, it is advised to use a low-WET transmission chamber in front of the water tank. The WET of this transmission reference chamber must be experimentally verified and accounted for.

Regarding the scanning direction of the detector, IDDs can be measured both horizontally (with the gantry at 90°) or vertically (gantry at 0°). Horizontal scanning has the advantage of a constant water pressure on the entrance window of the large-area ionization chamber along the whole scanning range. Thus, in case of entrance window deflection, that would be constant along the scan and it would therefore not affect the relative measurement. Moreover, the measurement is not affected by potential water surface ripples caused by the movement of the chamber and its holder. On the other hand, vertical scanning prevents the beam from going through the lateral wall (or entrance window) of the water phantom. This results in a better characterization of the IDD entrance region and, furthermore, it prevents the introduction of a systematic error in the determination of the range in water due to the uncertainty in the phantom wall thickness. No specific recommendation is given here regarding the direction of scanning. For horizontal scanning, the WET of the water phantom wall/entrance window must be experimentally verified and accounted for. For vertical scanning, it is recommended to scan from bottom to top to minimize the water surface ripples, as well as checking regularly the water level, which might slowly decrease due to water evaporation.

If possible, it is recommended to place the front face of the water phantom (horizontal scanning) or water surface (vertical scanning) at isocentre. If not possible, the reader must be aware that every 10 cm of displaced air corresponds roughly to a 0.1 mm overestimation of the range in water. Moreover, the large-area ionization chamber should be mounted parallel to the phantom wall/water surface and precisely centred on the beam central axis along the whole scanning range.

Theoretically speaking, ion recombination and ionization chamber response could vary with depth (i.e. along the scanning direction), as both dose rate and beam quality do so. However, both effects are expected to be minimal and have a negligible impact on the determination of *R_80_*. The ion recombination effect is easy to assess by simply acquiring two IDD curves at different polarization voltages—it has been reported to be negligible [32]. The effect of the beam quality is more difficult to assess, mainly because large-area ionization chamber response data is very scarce [33]. However, given the slow variation of beam quality correction factors (*k_Q_*) with energy [18], [34], [35], [36], [37], one could safely assume that this effect has a minimal impact on the IDD curves and, therefore, a negligible impact on the determination of *R_80_*.

Regarding the measurement resolution in depth, these guidelines recommend a variable step size, with a finer resolution in those regions with a higher dose gradient. As a general rule, acquisition settings should be optimized in order to minimize the activation of water.

Smoothing or any other post-processing of the signal is not recommended. Smoothing, in particular, is especially inadvisable, as it lowers the intensity of the maximum of the IDD curve and, therefore, it shifts the position of the 80 %-point beyond the Bragg peak that defines the range (*R_80_*).

**Fig. 4:**
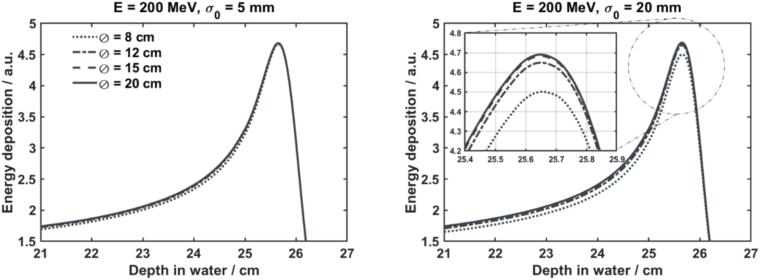
Integrated depth-dose curves of a 200 MeV proton pencil beam for different ionization chamber integrating areas (diameter = 8, 12, 15 and 20 cm) and different initial sizes of the beam: σ = 5 mm (left) and σ = 2 cm (right). The legend (left) applies to both plots.

The integration area of the ionization chamber has an effect on the shape of the IDD curves—see Fig. 4. Ideally, the TPS should take the finite integration area of the ionization chamber into account when deriving the energy distribution of the beam. This is the preferred approach, as it minimizes the effect of potential inaccuracies in the modelling of proton transport on the final dose calculation. When this is not the case, the user should correct for this effect by means of Monte Carlo simulation. For this particular problem, any extensively-tested general-purpose MC code (e.g. Geant4, FLUKA, PENH) may be used, since differences between them are minimal [38]. It is important, however, to restrict the area of the MC scorer to a size that is representative of the patient anatomy. If the area is too large, the scorer includes the contribution of secondary photons and neutrons that typically escape the patient. This work recommends a scorer integration radius of 20 cm.

Note also that water density at room temperature is few per mille smaller than 1 g/cm^3^. The user should keep this in mind in case the TPS requests the IDDs in units of mass thickness (e.g. g/cm^2^)—as this might lead to a systematic error in range of up to 1 mm for the extreme case of a 230 MeV beam and a water temperature of 25° (*ρ*_w_ = 0.997 g/cm^3^).

In case the TPS requests absolute IDD curves (e.g. in units of Gy mm^2^/MU), relative IDDs curves have to be normalized, at a reference depth, by the dose-area product in water (*DAP_w_*)—see 6.3 Reference dosimetry below.

To determine/validate the range-shifter WET, IDDs should be measured with the range-shifter inserted in the beam path, at least for the lowest and highest deliverable energies. The range-shifter WET is then obtained by subtracting the range in water with and without the range-shifter inserted in the beam path ($\text{WET}=R_{80,\text{out}}-R_{80,\text{in}}$). To verify the (lateral) homogeneity of the range-shifter, it is advisable to repeat this procedure for different lateral positions of the beam, moving the centre of the large-area ionization chamber (or the whole water phantom) accordingly.

Finally, it is very common within the PT community to label the beam using its initial mean energy. It is important to remember that the beam label should be consistent amongst TDS, TPS and experimental data.

### 6.2 Spot maps in air

As mentioned above, TPSs ask for centred spot profiles in air at minimum 3 different *z*-positions. These guidelines, however, recommend acquiring not only single-spot profiles, but spot maps in air at 5 different *z*-positions^19^ for at least one reference gantry angle. Furthermore, it recommends verifying these spot maps, at least at isocentre, for different gantry angles.

The distance between the 5 measurement *z*-positions should be as large as possible to better characterize the initial moments of the ASD and to minimize the sensitivity of the fit to individual data points. These guidelines recommend an asymmetrical data acquisition around the isocentre, shifted towards the nozzle, to improve the characterization of the ASD in the clinically relevant range.

Moreover, measurements at different lateral (*xy*) positions and different gantry angles are recommended, as the beam optics may depend on these factors, resulting in notably different spot shapes. Ideally, the spot shape should be independent of the lateral position and the gantry angle and, as such, TPSs only accept one initial ASD model. However, this is not always the case in practice. If differences in spot shape exist, the user should introduce a value which is representative of all possible scenarios, keeping in mind that spot positions close to the isocentre are much more frequent than extreme spot positions at the edges of the maximum field size. Also, not all gantry angles are equally probable (although this could change in the future with the implementation of arc therapy). Typically, differences in spot size in air of +/− 10 % are considered acceptable [39], [40], [41].

Spot maps should be measured with a high spatial resolution 2D detector, such as radiochromic films, scintillating screens coupled to a CCD/CMOS camera or amorphous-silicon flat panel detectors [42]. Some of these detectors are not water-equivalent [43] and suffer from quenching [44], [45], [46], [47], but they are all suitable for relative measurements of 2D dose distributions in air. Acquisition parameters should be optimized in order to maximize the signal-to-noise ratio without causing detector saturation.

Another important thing to consider when acquiring spot maps is the settling time of the beam line magnets after an energy change. It is crucial that the beam position is stable when acquiring the data, otherwise the measurement will suffer from a blurring artefact. The users can estimate the magnets settling time either by acquiring the data in video mode (provided the time resolution of the detector is fast enough) or by inspecting the machine log-files.

Finally, TPSs also allow to create a double-Gaussian model of the spots that improves the modelling of the low-dose tails [48]. If the dynamic range of the user detector is not sufficient to obtain a good signal-to-noise ratio in the low-dose tails without saturating in the central part of the spot, the user could follow the approach in Ref. [49]. That is, to acquire two spot maps per measurement: one with a low dose to characterize the primary beam and a second one with a high dose (causing detector saturation at the central part of the spots) to obtain a high SNR in the low-dose tails.

### 6.3 Reference dosimetry

These guidelines recommend the adherence to an international radiation dosimetry protocol/code of practice. In particular, it recommends the adherence to the recently published revision of the IAEA TRS-398 [18]—the only international code of practice that includes scanned proton beams.

IAEA TRS-398 addresses the determination of the absorbed dose to water (*D_w_*). If needed, *D_w_* at the centre of a broad field can be converted to the dose-area product in water (*DAP_w_*) of a single pencil beam using [33]:

$${\mathrm{DAP}}_{w}≅D_{w}\Delta_{x}\Delta_{y}$$

provided the distance between adjacent spots at the measurement depth (Δ_x_, Δ_y_) is sufficiently small to generate a uniform dose distribution across the broad field.

These guidelines recommend an independent validation of the output. Ideally, by means of an external audit. Alternatively, using a completely independent dosimetry method, such as calorimetry or Faraday cup dosimetry—although this alternative may not be feasible for most centres. Else, dosimetry inter-comparisons between peers are also useful and typically easier to carry out. Last, the output should be verified at different gantry angles.

### CRediT authorship contribution statement

**Carles Gomà:** Conceptualization, Methodology, Investigation, Writing – original draft, Visualization, Project administration. **Katrin Henkner:** Conceptualization, Investigation, Writing – review & editing. **Oliver Jäkel:** Conceptualization, Investigation, Writing – review & editing. **Stefano Lorentini:** Conceptualization, Investigation, Writing – review & editing. **Giuseppe Magro:** Conceptualization, Investigation, Writing – review & editing, Visualization. **Alfredo Mirandola:** Conceptualization, Investigation, Writing – review & editing, Visualization. **Lorenzo Placidi:** Conceptualization, Investigation, Writing – review & editing. **Michele Togno:** Conceptualization, Investigation, Writing – review & editing. **Marie Vidal:** Conceptualization, Investigation, Writing – review & editing. **Gloria Vilches-Freixas:** Conceptualization, Investigation, Writing – review & editing, Visualization. **Jörg Wulff:** Conceptualization, Investigation, Writing – review & editing. **Sairos Safai:** Conceptualization, Investigation, Writing – review & editing, Visualization.

## Declaration of competing interest

The authors declare that they have no known competing financial interests or personal relationships that could have appeared to influence the work reported in this paper.

## Footnotes

14 Eclipse also requires these data with at least the thickest range-shifter inserted.

16 Eclipse requests the nominal SAD, so no experimental measurement is needed.

17 RayStation requests the range-shifter(s) material, density and physical thickness.

## Footnotes

12 These guidelines is based on versions RayStation 12A [14], [15] and Eclipse 18.0 [16].

13 In these guidelines, the term ‘pencil beam’ refers to a ‘finite pencil beam’ [20]. See Table 1.

15 Note that only those beamline parameters that need to be determined by means of an experimental measurement are included in this report.

18 Mevion S250i HYPERSCAN.

19 These guidelines define the *z*-axis as the beam propagation axis. Please note that the TPS coordinate system may differ from this definition.
